# Machine Learning‐Based Identification of Petroleum Distillates and Gasoline Traces Using Measured and Synthetic GC Spectra from Collected Samples

**DOI:** 10.1002/minf.70008

**Published:** 2025-08-22

**Authors:** Omer Kaspi, Yaniv Y. Avissar, Arnon Grafit, Ron Chibel, Olga Girshevitz, Hanoch Senderowitz

**Affiliations:** ^1^ School of Electrical Engineering – System Engineering Program Afeka College of Engineering Tel Aviv Israel; ^2^ School of Industrial Engineering and Management Shenkar College of Engineering Design and Art Ramat‐Gan Israel; ^3^ Ignitable, Poisons and Explosives Lab Israel Police HQ Jerusalem Israel; ^4^ Bar Ilan Institute of Nanotechnology and Advanced Materials Bar‐Ilan University Ramat‐Gan Israel; ^5^ Department of Chemistry Bar‐Ilan University Ramat‐Gan Israel

**Keywords:** classification, data synthesis, deep learning, forensic informatics, gasoline, ignitable liquids, machine learning, petroleum distillates

## Abstract

Ignition cases involving arsons are typically handled by forensic experts who examine spectra of samples collected from scenes of fire to test for the existence or absence of ignitable liquids. This is tedious work, since many cases do not involve such liquids. To facilitate this process, we have developed in this work a Machine Learning (ML)‐based workflow for samples’ classification based on their gas chromatography (GC) chromatograms (i.e., spectra). To this end, annotated spectra of 181 samples containing three groups of liquids (petroleum distillates, gasoline, and an assortment of other substances) collected from fire scenes as well as two reference databases were obtained from the Israeli Department of Identification and Forensic Sciences (DIFS). These spectra were used for the derivation of ML‐based classification models using three algorithms, namely, *k*NN, representative spectrum, and random forest (RF) giving rise to reliable predictions. To increase the size of the dataset to a level that would enable the usage of more advanced ML algorithms, we have used the experimental spectra to develop a new spectra synthesis algorithm and utilized it to generate a large dataset of synthetic spectra. This dataset was used for the derivation of new *k*NN, RF, and representative spectrum models as well as deep learning (DL) models producing F1‐scores over an independent test set composed entirely of “real” spectra ranging from 0.74–0.95, 0.86−0.95, 0.30–0.75, and 0.85–0.96 for *k*NN, RF, representative spectrum, and DL, respectively. Following the completion of the work, a second set of real spectra was provided to us by DIFS, and modeling it with the second set of models yielded F1‐scores ranging from 0.92–0.96, 0.96−1.00, 0.71–0.82, and 0.95–0.98 for *k*NN, RF, representative spectrum, and DL, respectively. These results therefore suggest that for this dataset, performances depend more on the size of the dataset used for model training than on the ML algorithm. We propose that the workflow and spectra synthesis algorithm developed in this work could be readily applied to other forensic domains where samples are characterized by spectra, either solely or in combination with other parameters.

## Graphical abstract

In this work, we used a combination of real and synthetic gas chromatography (GC) spectra to develop an machine learning (ML)‐based workflow for the classification of flammable liquids into gasoline, petroleum distillates, and other liquids. The resulting models demonstrated high classification accuracy on two external test sets.

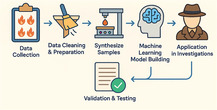

## 1 Introduction

Current studies show that the vast majority of currently investigated arson cases involve the usage of petroleum distillates as well as gasoline [1, 2]. These materials are of special importance to law enforcement agencies as they are highly accessible, affordable, and highly volatile and do not require any expertise to ignite and cause significant damage to both lives and property. In cases that involve arson, analytical labs of law enforcement agencies typically employ highly proficient and skilled personnel to measure, evaluate, and determine the presence of these materials within samples found in crime scenes.

For this analysis, the forensic evidence (exhibit) is collected at the scene of the fire and sent for laboratory examination [3] with the aim to determine whether an ignitable liquid (IL) was used [4]. One of the analytical procedures used by forensic laboratories for the detection and classification of IL residues is headspace, a solid phase microextraction gas chromatography mass spectrometry technique (HS‐SPME /GC–MS [5, 6, 7–8]).

Yet, despite analytical improvements, the analysis of fire debris and their interpretation is still a time‐consuming and qualitative process, which mostly relies on the expertise and experience of the forensic expert [9]. To aid this important endeavor, the usage of machine learning (ML) and in particular various classification algorithms has been growing steadily in the past years.

Much of the work in this field was performed by Sigman et al., who published numerous papers dealing with the identification and classification of IL within samples based on their GC/MS spectra [10, 11, 12, 13, 14, 15–16]. These works are based on neat IL spectra as well as on samples retrieved from controlled fires (i.e. a fire was set within a “furnished” container to simulate a real fire). As part of these studies, a dataset of in silico generated total ion chromatogram (TIC) spectra was developed and used for the training of some of the ML models. Data were visualized and clustered using dimension reduction methods such as principal component analysis (PCA) and self‐organizing feature maps (SOFMs) while models were developed using various classification methods such as naïve Bayes, linear discriminant analysis (LDA), quadratic discriminant analysis (QDA), support vector machine (SVM), *k*‐nearest neighbors (*k*NN), and several neural networks. The main purpose of all ML models was to classify samples as IL containing or not IL containing often by allocating a “strength of evidence” measure to the predictions based on the calculation of log likelihood ratios. Most recently Sigman et al. [17] have published a convolutional neural network (CNN)‐based model for the classification of samples as IL containing/not IL containing. The performances of this model were evaluated using the area under the receiver operating characteristic curve (ROC‐AUC) metric and led to values of 0.87 and 0.99 for test sets containing laboratory‐generated fire debris samples and a combination of neat IL and single‐substrate samples, respectively. Once an IL was classified, the researchers attempted to identify the type of IL obtaining success rates ranging from 45 to 82.3%. A subsequent paper by the same group combined ML models with human analysis into a consensus model obtaining ROC AUC values of 0.90–0.95 [18]. Most recently, the synthetic dataset developed by the Sigman lab was extended to include 240,000 TIC spectra and used to train an XGBoost model which, when validated on experimental fire debris data achieved an ROC‐AUC of 0.845 [19].

Additional contributions in this field were made by Elmaz et al. who have used ML to classify different types of solid fuels achieving a correct classification rate of 92% [20], Pasternak et al. [21], who derived a rule‐based classification model with high performances [21], and Bogdal et al. [22, 23] who described various methods to identify the existence of gasoline in fire debris. Finally, Huang and Yu leveraged transfer learning of pre‐trained CNNs on transformed (into three types of image presentations) GC–MS data for improved classification in fire debris analysis. The resulting models achieved 100% accuracy in identifying neat gasoline and 95.9% accuracy in identifying gasoline residues in spiked samples [24].

In sequel to these previous efforts, in this work, we developed several ML‐based models for the classification of samples into petroleum distillates, gasoline, and other flammable liquids‐containing, based on the gas chromatography (GC) component of their gas chromatography–mass spectrometry (HS‐SPME /GC–MS) spectra. To this end, we first used a set of ∼180 annotated samples provided to us by the Israeli DIFS. A set of this size allowed us to develop predictive models based on the *k* nearest neighbors (*k*NN), random forests (RF), and representative spectrum methods, yet it was insufficient for developing more advanced models, e.g., based on deep neural networks (also known as deep learning (DL)). Thus, we developed a new algorithm, based on physical principles, to generate a large dataset of synthetic spectra and used them to develop a DL‐based model and for comparison, new *k*NN, RF and Representative Spectrum‐based models. These models were first evaluated on test sets composed entirely of real spectra and subsequently, on a second test set of real spectra provided to us by DIFS following the completion of the work, leading, in most cases, to reliable predictions for both datasets.

## 2 Materials and Methods

### 2.1 Datasets

In this work, we used four datasets, all provided to us by the Israeli DIFS. (1) First is an annotated dataset consisting of 181 samples collected from ‘real’ (i.e., not controlled) fire scenes investigated by the Israel Police during 2017–2021. Samples of this set were annotated by two independent arson forensic experts by visually comparing their spectra to reference chromatograms using the overlay Agilent Chemstation and Agilent MassHunter software in TIC and single ion modes. Following this analysis, it was concluded that the samples are composed of petroleum distillates (PD, 81 cases), gasoline (BZ, 53 cases), or any other flammable substance (HR, 47 cases; the most common components in this class were acetone and ethanol). This database was used for model training and evaluation and for constructing synthetic spectra (see below). (2) Second is a similarly annotated dataset, provided to us after the completion of the work and consisting of 89 samples (31 PD, 32 BZ, and 26 h) collected by DIFS from cases investigated during 2022. This dataset was used for further validation of the models. (3) Third is a reference dataset containing 13 samples of gasoline, taken from various commercial brands from local gas stations and evaporated to 0%, 20%, 50%, 60%, 70%, 80%, 90%, 95%, and 99%. (4) Fourth is a second reference dataset containing 17 samples of petroleum distillates, also taken from various commercial brands, where diesel fuels were evaporated to 0% and 25%, and kerosene was evaporated to 0% and 75%. Similar to the first dataset, the two reference datasets were also used for model training and validation and for generating the synthetic spectra.

Reference and casework samples were placed in sealed nylon bags or glass vials and transported on the same day, in ambient temperature, to the Arson Investigation Laboratory. The samples received for analysis in the laboratory arrived either in liquid form in a 4 mL glass vial (∼10% of the samples) or in a solid form in a sealed nylon arson evidence bags 460, 9600, and 0.04 mm thick from Grand River Products, LLC (Grosse Pointe Farms, MI). Since most samples were stored in nylon bags and only for several hours, expansion or pressurization of the bags was not a problem. Samples were heated for 15 min at 130°C, and then 5 mL of gas was taken with a syringe or extracted during 15 min with solid phase microextraction fiber (SPME fiber assembly polydimethylsiloxane (PDMS) df 100 μm, needle size 24 gauge, for use with manual holder, purchased from Supelco) under ambient temperature by inserting the fiber assembly into the HS of the nylon bag, in accord with the ASTM E2154−01 standard [5].

Controlling for carry‐over in solid phase microextraction (SPME) is crucial to ensure accurate and reproducible results, especially in trace analysis. Carry‐over refers to residual analytes from a previous sample that remain on the SPME fiber and affect the subsequent analysis. In this work, we used multiple methods to minimize carry‐over. (1) The analytical method is optimized so that desorption time and temperature are sufficient to avoid carry‐over. The fiber is exposed to 290°C for 10 min in the GC injector to ensure complete release of analytes. (2) After, each extraction/desorption cycle fibers were conditioned in a separate GC injector. (3) In order to eliminate residual contamination from the previous sample, a blank‐run was analyzed. (4) A coating with appropriate affinity for the target was used to reduce the chance of residuals remaining. This procedure ensures better sensitivity and reduces false negative rate in comparison with lower temperatures, and in addition, it is more time efficient and cost‐effective in comparison with the standard defined by ASTM 1618 [25].

Samples were injected into the GC–MS injector of an Agilent technologies 7890A gas chromatograph coupled with an Agilent Technologies 5975C inert XL MSD quadrupole mass spectrometer. The injector temperature was 280°C, and the injection was made in a split mode (30:1). A 30 m HP‐5 MS column was used with 0.25 mm internal diameter and 0.25 μm stationary phase thickness. Helium (99.999%) mobile phase flowed at a flow rate of 1.2 mL/min, which may slightly reduce the resolution but uses less helium [21]. The oven temperature program was set to 1 min at 35°C followed by heating to 230°C at 15°C/min and then heating to 280°C at 25°C/min and holding the temperature constant for 1.5 min. Ionization was performed using standard EI source applying 70 eV at 230°C. The transfer line and the quadruple temperature were set to 270°C and 150°C respectively. Mass scanning range at 0–2 min was 30–100 amu (which is sufficient for light compounds, while eliminating background noise from air) and then 25–350 amu.

### 2.2 Chromatogram (Spectra) Preprocessing

**FIGURE 1:**
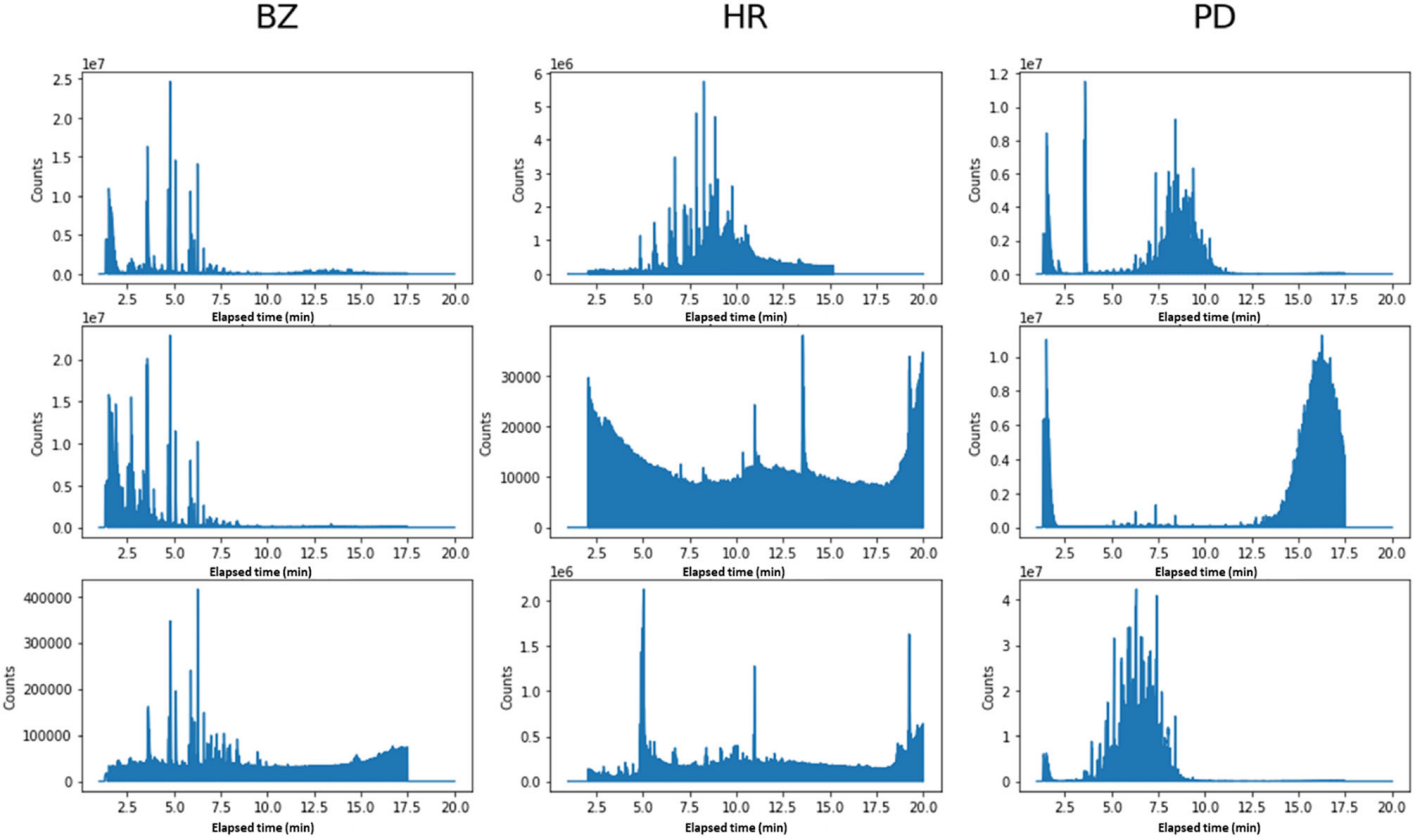
Examples of digitized spectra of BZ, HR, and PD. GC chromatograms may show variability depending on multiple factors. For BZ, the three chromatograms differ by their intensities; for HR, the three chromatograms differ by having backgrounds from different substrates; and for HR, the three chromatograms differ by the levels of weathering conditions.

Prior to model construction, the GC chromatograms, expressed in terms of intensity vs. time, were digitized into 2924 bins of 0.4 s (the measuring device's resolution) covering the entire measurement time from 1 to 20 min. Measurements within a single 0.4 s interval were summed into a single value. Figure 1 presents examples of digitized spectra for BZ, PD, and HR.

Next, the resulting spectra were randomly partitioned into training and test sets in a 67%/33% ratio. The former set was used to train the classification model while the latter set was held back from the training process for an unbiased performance evaluation (Table 1).

**TABLE 1:** Per class numbers of samples in training and test sets.

	Dataset	Training set	Test set
PD	81 (44.7%)	54 (44.6%)	27 (45.0%)
BZ	53 (29.2%)	36 (29.7%)	17 (28.3%)
HR	47 (25.9%)	31 (25.6%)	16 (26.6%)

### 2.3 Synthetic Spectra

**FIGURE 2:**
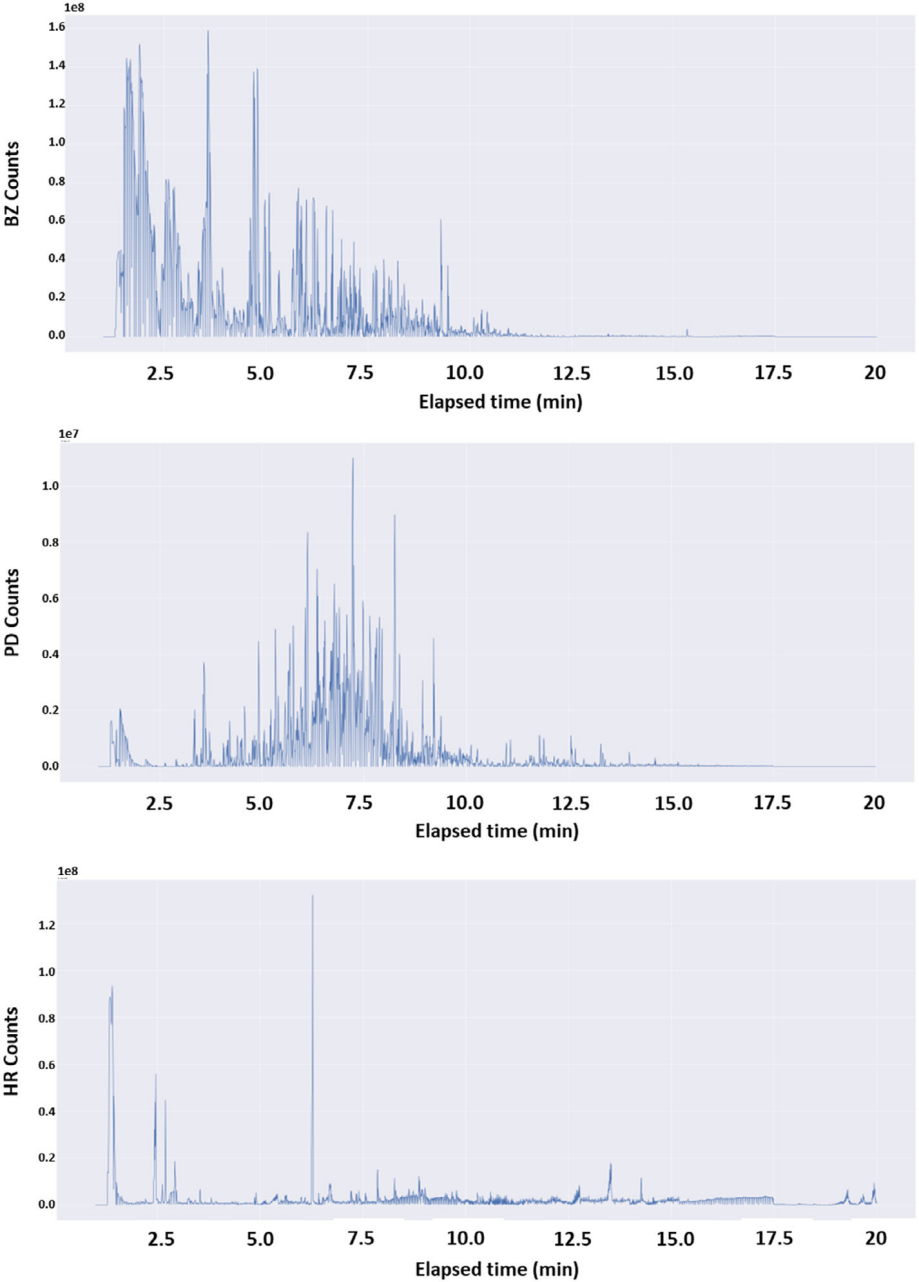
A synthetic spectrum for BZ (top), PD (middle), and HR (bottom).

In principle, the performances of ML algorithms improve with the size of the dataset available for training. While not all algorithms require very large datasets to be trained on, neural network (NN)‐based algorithms do. Due to the relatively small size of our dataset, we therefore developed a new procedure for generating synthetic spectra. Our basic hypothesis in this process was that linearly combining spectra of samples from the same class will lead to a plausible spectrum characteristic of this class (e.g., ‘gasoline + gasoline = gasoline’). Based on this hypothesis, synthetic spectra were generated using a two‐step, draw and combine procedure. In the draw phase, a random number (*k*) between one and the total number of samples from a specific group (BZ, PD, or HR) within the training set (*N*) was generated. Next, *k* sample‐vectors were randomly drawn from the training set thereby creating a *k* by 2924‐dimension matrix (will be referred to as *X*). The number of possible matrices is given by $\sum_{k=1}^{N}\frac{k!}{k!\left(N-k\right)!}$ amounting to an extremely large number of combinations (>>10^50^). Next, in the combine phase, a random weight between −4 and 4 was generated for each sample‐vector in *X* leading to a weights vector of length *k* (termed *W*). Finally, a dot product between *W* and *X* produced a new vector (i.e., spectrum) as a weighted linear combination of the drawn *k* spectra. Overall, 10^5^ synthetic spectra were constructed using this procedure. Figure 2 presents an example of a synthetic spectrum of each class.

To test the plausibility of the spectra synthesis method, we have challenged two forensic experts from DIFS with a mixture of real and synthetic spectra and asked them to differentiate between the two types (i.e., real or synthetic) and to classify the spectra to one of the three spectra classes (i.e., BZ, PD, and HR). The experts performed visual inspection of 24 real and 76 synthetic chromatograms using their own expertise and based on known profiles of gasoline and PD. In this analysis, they did not consider the MS information. We chose this approach since model development also considered only the GC component of the samples.

### 2.4 Data Normalization

Normalizing the data prior to deriving ML models is important in order to prevent numerical values from biasing the model derivation process. For DL networks, normalization of the data to values between −1 and 1 is mandatory. When values (inside the sample's vector) are above or below this range, it is likely that most activation functions (the nonlinear operations) will change very little and thus the training will get ‘stuck’. While there are many ways to perform normalization, due to the large range of potential values (typically between 10^5^–10^7^), the following normalization was implemented (Equation (1))

(1) $$\text{normalized value}=\frac{\log\;\left(\text{value}\right)}{\text{upper}\log\;\text{bound}-\text{lower}\log\;\text{bound}}+\text{lower}\log\;\text{bound}$$

**FIGURE 3:**
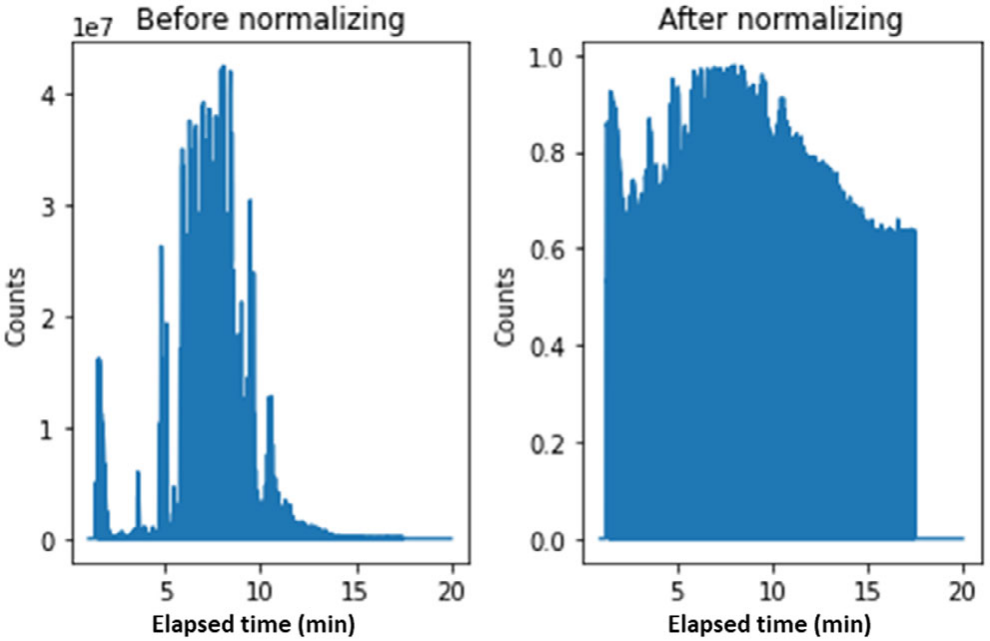
A spectrum of evaporated BZ before (left) and after (right) normalization.

First, the intensities of training set chromatograms were converted to their log values, to handle their large range covering several orders of magnitude (a consequence of the unique characteristics of the different crime scenes). Then, the maximal and minimal values of each bin were recorded (for future application on the test set) and used to normalize the data to the [0–1] range. Since normalization only considered the training set, test set data may be converted to values outside this range. In such cases, values above 1 or below 0 were set to 1 and 0, respectively. Figure 3 presents a spectrum before and after normalization.

### 2.5 Model Derivation

Using Python 3.8 and standard SciKit modules, four types of classification models were developed: *k*NN, Distance from a representative spectrum, RF, and DL. The first three algorithms could be trained using a small number of samples, and consequently respective models were derived using both the real spectra and the combination of real and synthetic spectra. DL models could only be trained on a large set of spectra, and consequently, only the combination of real and synthetic spectra was used for their training.

*k*NN is perhaps the most intuitive method of classification. It classifies a new, unlabeled, sample by comparison to a set of known, labeled, samples (a training set). Thus, the model labels an unknown sample by identifying the class that is most prominent among its *k* nearest neighbors. The value of *k* was optimized to the value of 3 using 3‐fold cross‐validation (CV), with 10 iterations on the training set.

**FIGURE 4:**
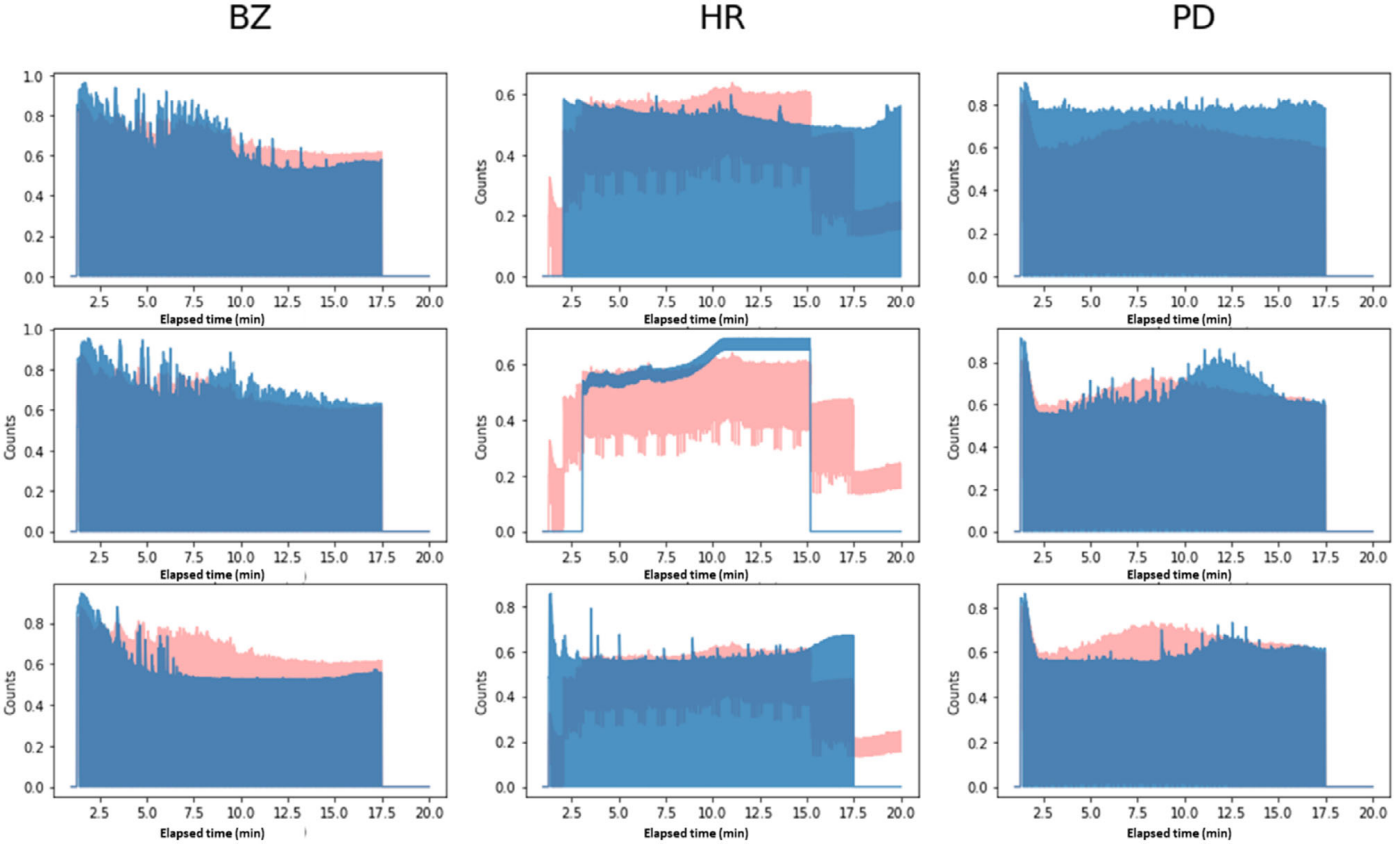
In columns, spectra labeled as BZ, PD, and HR. In red, the representative spectrum of the class, and in blue, spectra of individual samples.

Classification based on distance from a representative spectrum assumes that each class has a representative form (i.e., spectrum) that could be approximated as the mean of many samples (i.e., spectra) that belong to that class. Figure 4 demonstrates this idea by presenting individual spectra overlaid on a representative spectrum of all three classes. An unknown sample's class is determined using the label of the representative spectrum most similar to it. In this work, both *k*NN and representative spectra used the Euclidean distance in spectral space.

RF is another ML algorithm that could be used either for classification or for regression. RF is composed of a group of single classifiers called trees. Each tree could be regarded as a set of consecutive rules [26], and each rule divides the dataset into smaller groups until an end criterion is met. Each tree classifies each sample into one of pre‐determined classes and the final classification is determined by a majority vote. A new unknown sample will be evaluated using these sets of rules and its label will be determined by the group it falls into. The development of RF models requires the optimization of several hyperparameters [26]. In this work, optimization of hyperparameters was independently preformed for the two models constructed in this work (based on real data only or on a combination of real and synthetic data) using a grid search and 3‐fold 10 iterations CV. Table 2 presented the hyperparameters optimized in this work as well as their final values.

**TABLE 2:** RF parameter optimized in this work. Optimization was performed using a grid search and 3‐fold, 10 iteration CV.

Parameter	Description	Search span	Tuned value
Num_estimators	Number of different trees in the forest	Min value: 200 Max value: 1800 Step size: 200	200
max_features	The maximum allowed number of features to consider for the best possible split	max_features = log2 (n_features) or max_features = sqrt (n_features)	max_features = sqrt (n_features)
max_depth	The maximum depth of the tree	Min value: 10 Max value: 100 Step size: 10	No Max depth limitation
min_samples_split	The minimum number of samples required to split an internal node	2, 5 or 10	2
min_samples_leaf	The minimum number of samples required to be at a leaf node	1, 2 or 4	1
bootstrap	Whether bootstrap samples are used when building trees	True or False	True

**FIGURE 5:**
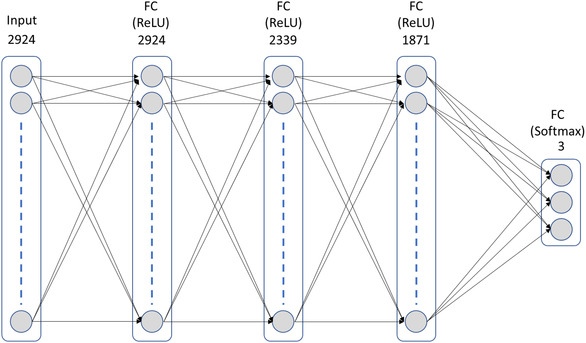
Schematic representation of the neural network used in this work with an input layer, three hidden layers, and an output layer with three classes.

The most complex classification model used in this work is DL. In this work, we have implemented a fully connected NN with three hidden layers, each activated by a rectified linear unit (ReLU), three output nodes, one for each class, and a softmax activation function (Figure 5). Network parameters were optimized using Adam optimizer leading to a learning rate of 10^−5^ and to the usage of a sparse categorical cross entropy loss function.

### 2.6 Model Evaluation Metrics

**FIGURE 6:**
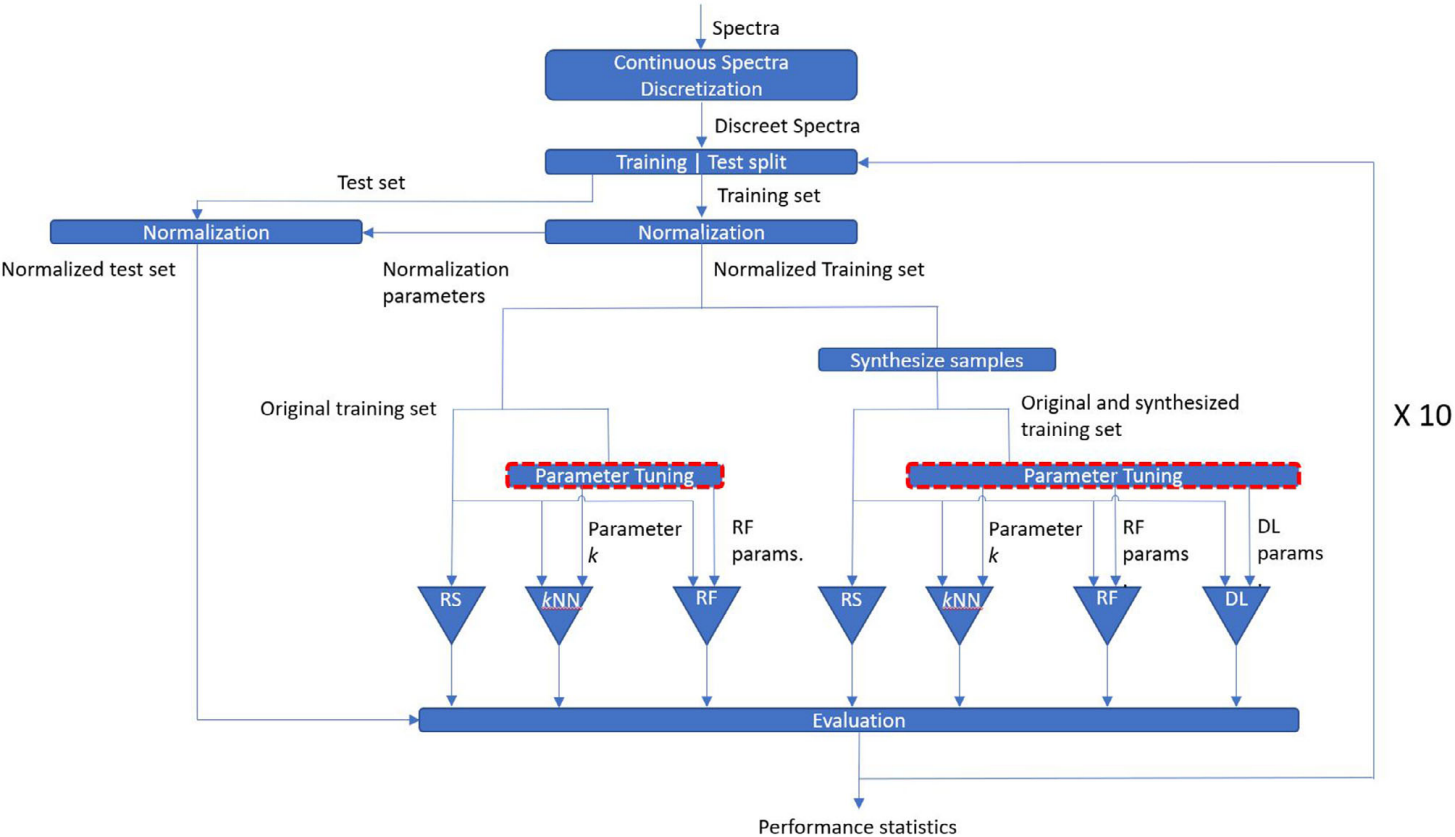
The ML workflow used in this work with ten repetitions for statistical evaluation.

Model performances were evaluated by three metrics: recall, precision, and F1‐score which together allow for a complete evaluation of model performances. Recall (Equation (2)) indicates the model's ability to effectively identify a class by considering both true positive (TP) and false negative (FN). Precision (Equation (3)) indicates the model's ability to correctly determine a TP. F1‐score (Equation (4)) is the harmonic mean of precision and recall and provides a balance of the two. To get reliable statistical estimates for model performances, the entire procedure was repeated ten times, each time using a different training/test split (see Figure 6).

(2) $$\text{Recall}=\frac{\mathrm{TP}}{\mathrm{TP}+\mathrm{FN}}$$

(3) $$\text{Precision}=\frac{\mathrm{TP}}{\mathrm{TP}+\mathrm{FP}}$$

(4) $$F1-\text{Score}=\frac{\text{Recall*Precision}}{\left(\text{Recall}+\text{Precision}\right)*2}$$

## 3 Results

A first set of *k*NN, RF, and representative spectrum‐based models was derived and validated using the real data only (see Table 1), and the results are presented in Table 3. Both *k*NN and RF generated models with overall good performances across all classes and a good balance between precision and recall as reflected in their F1‐scores. In contrast, poorer results were obtained with the representative spectrum algorithm.

**TABLE 3:** Test set performances of *k*NN, RF, and representative spectrum classification models trained on real data only. Results are reported as average ± SD over ten random splits (see Materials and Method section for more details).

***k*NN**			
	**Precision**	**Recall**	**F1‐score**
BZ	0.75 ± 0.09	0.81 ± 0.11	0.77 ± 0.08
PD	0.85 ± 0.05	0.83 ± 0.07	0.83 ± 0.05
HR	0.84 ± 0.10	0.90 ± 0.09	0.87 ± 0.07

**RF**			
	**Precision**	**Recall**	**F1‐score**
BZ	0.85 ± 0.08	0.84 ± 0.08	0.84 ± 0.07
PD	0.88 ± 0.07	0.85 ± 0.05	0.86 ± 0.05
HR	0.90 ± 0.07	0.95 ± 0.05	0.92 ± 0.05

**Representative spectrum**			
	**Precision**	**Recall**	**F1‐score**
BZ	0.84 ± 0.11	0.55 ± 0.12	0.65 ± 0.10
PD	0.76 ± 0.08	0.51 ± 0.09	0.61 ± 0.08
HR	0.52 ± 0.05	0.99 ± 0.03	0.68 ± 0.04

*k*NN and RF produced models with good predictive statistics; however, we wanted to see whether even better models could be obtained using the DL method. Since DL requires a large dataset for the training process, we had to rely on synthetic data for model training. However, prior to using the synthetic data, we needed to ascertain that these are plausible representations of the different arsons. To this end, we have conducted a blind test whereby we have challenged two experts from DIFS with a mixture of real and synthetic spectra and asked them to (a) differentiate between the two types and (b) classify the spectra to one of the three classes. The experts managed to correctly determine whether the spectrum is real or synthetic in only 60% of the cases (suggesting that the experts found the real and synthetic spectra rather indistinguishable), yet in almost all cases, they were able to correctly classify the synthetic spectra into one of the three classes.

Having demonstrated the plausibility of our synthetic spectra, we have used them, in combination with real data, to train a DL classifier. Importantly, the performances of the resulting classifier were evaluated on a test set of real data only (Table 4).

**TABLE 4:** Test set performances of the DL model, trained on a combination of real and synthetic spectra, and evaluated on real data only. Results are reported as average ± SD over ten random splits.

**DL**			
	**Precision**	**Recall**	**F1‐score**
BZ	0.79 ± 0.11	0.92 ± 0.05	0.85 ± 0.07
PD	0.92 ± 0.05	0.81 ± 0.11	0.86 ± 0.07
HR	0.96 ± 0.03	0.96 ± 0.06	0.96 ± 0.04

The small improvement in performance of the DL model in comparison with the other three models could be attributed to the larger dataset used for its training. Thus, we have repeated the training process for the other three algorithms this time using the combination of real and synthetic data for model training.

As before, models were evaluated on test sets composed of real data only (see Table 5). A comparison between the current results and those reported in Table 3 indicates that the performances of *k*NN roughly remained the same, the performances of RF slightly improved across all metrics, and the performances of the representative spectrum algorithm displayed a significant improvement in one metric (recall or precision) while suffering from a substantial performance degradation in the other (see discussion below).

**TABLE 5:** Test set performances of *k*NN, RF, and representative spectrum models trained on a combination of real and synthetic spectra and evaluated on real data only. Results are reported as average ± SD over ten random splits.

***k*NN**			
	**Precision**	**Recall**	**F1‐score**
BZ	0.70 ± 0.06	0.77 ± 0.09	0.74 ± 0.07
PD	0.88 ± 0.06	0.77 ± 0.06	0.83 ± 0.05
HR	0.91 ± 0.06	1.00 ± 0.00	0.95 ± 0.03

**RF**			
	**Precision**	**Recall**	**F1‐score**
BZ	0.84 ± 0.08	0.89 ± 0.06	0.86 ± 0.06
PD	0.93 ± 0.04	0.87 ± 0.07	0.90 ± 0.05
HR	0.94 ± 0.06	0.97 ± 0.03	0.95 ± 0.04

**Representative spectrum**			
	**Precision**	**Recall**	**F1‐score**
BZ	0.38 ± 0.21	1.00 ± 0.00	0.55 ± 0.22
PD	1.00 ± 0.00	0.15 ± 0.07	0.30 ± 0.10
HR	0.98 ± 0.04	0.61 ± 0.07	0.75 ± 0.06

As noted in the Methods section, following the completion of the work, an additional dataset of 89 samples (32 BZ, 26 h, and 31 PD) was provided to us by DIFS and used for further validation of the models derived from the combination of real and synthetic spectra. This new dataset was normalized using the same normalization parameters derived from the original dataset. Models’ performances on this dataset are presented in Table 6.

**TABLE 6:** Performances of the DL, *k*NN, representative spectrum, and RF models on the additional dataset. Results are reported as average ± SD over ten random splits.

**DL**			
	**Precision**	**Recall**	**F1‐score**
BZ	0.96 ± 0.05	0.99 ± 0.01	0.97 ± 0.02
PD	0.98 ± 0.01	0.92 ± 0.07	0.95 ± 0.04
HR	0.96 ± 0.02	1.00 ± 0.00	0.98 ± 0.01

***k*NN**			
	**Precision**	**Recall**	**F1‐score**
BZ	0.92 ± 0.01	0.94 ± 0.03	0.93 ± 0.01
PD	0.94 ± 0.02	0.89 ± 0.03	0.92 ± 0.02
HR	0.96 ± 0.03	1.00 ± 0.00	0.96 ± 0.02

**RF**			
	**Precision**	**Recall**	**F1‐score**
BZ	0.96 ± 0.04	0.97 ± 0.02	0.97 ± 0.02
PD	0.97 ± 0.02	0.96 ± 0.04	0.96 ± 0.02
HR	1.00 ± 0.00	1.00 ± 0.00	1.00 ± 0.00

**Representative Spectrum**			
	**Precision**	**Recall**	**F1‐score**
BZ	0.84 ± 0.03	0.80 ± 0.01	0.82 ± 0.02
PD	0.62 ± 0.01	0.84 ± 0.03	0.71 ± 0.02
HR	1.00 ± 0.00	0.65 ± 0.00	0.79 ± 0.00

## 4 Discussion and Conclusions

Using ML for the analysis of fire debris is an active field of research with important forensic applications. Prior studies in the field, primarily from the Sigman lab, mainly focused on classifying controlled samples into IL‐containing and non‐IL‐containing with notable successes. However, when some of these models were applied to a dataset containing mixtures of neat IL with controlled fire debris, performances in terms of true positive rate were low (45%) [17]. Guided by the operational needs of DIFS, in this work, we set up to develop ML models for a multiclass classification of samples into petroleum distillates, gasoline, and an ‘other’ category, using ‘real‐life’ fire debris samples as opposed to controlled samples. In doing, so we hope to bring such models closer to practicality. Other differences from previous studies are the smaller dataset we used (a consequence of using real data as opposed to controlled data) and reliance on the GC component of the samples’ GC–MS spectra as opposed to using the TIC of the different samples. This also required the usage of a different method for data synthesis (i.e., a linear combination of multiple GC chromatogram).

Overall, in this work, we derived several classification models for three classes of ignitable liquids, namely, petroleum distillates, gasoline, and an assortment of other substances. Since the performances of ML‐based models are known to improve with the size of the dataset used for their training, and due to the specific training set size requirements of DL models, we have devised a new procedure for synthesizing synthetic spectra. In this work, we chose to construct synthetic spectra by linearly combining multiple spectra in order to eliminate the potential effect of ‘irregular’ spectra. This is similar to the approach used, for example, by the RamansSPy library [27]. Furthermore, in order not to predefine the “right number” of spectra to be combined, this number was selected at random. The weights were selected to ensure that no single spectrum (or spectra) dominates all other spectra when linearly combined, yet the exact range ([−4, 4]) was somewhat arbitrarily selected after evaluating multiple ranges that all led to similar results. Importantly, based on expert evaluation, we found the synthetic spectra to be almost indistinguishable from real spectra. Thus, DIFS experts were able to correctly classify spectra as real or synthetic only in 60% of the cases, not very different from a random classification.

Next, we used either real or real+synthetic spectra for model derivation. We found that increasing the size of the training set by including synthesized spectra had different effects on the performances of different algorithms. Thus, *k*NN displayed improvements in some of the metrics yet its performances were not largely affected by the larger dataset used for training. The performances of the RF algorithm improved across all metrics (albeit not significantly). Finally, the representative spectrum algorithm displayed a significant improvement in precision coupled with a significant decrease in recall for the PD and HR classes and an opposite effect for the BZ class, with no clear trend in F1‐Score. We attribute the large effect of training the representative spectra‐based model with synthetic spectra on its performances, to the fact that representative spectra for all three groups of flammable liquids were generated by averaging individual spectra and are therefore more sensitive to ‘extreme’ spectra that could have been generated by the data synthesis algorithm. However, at this stage, we are unable to rationalize the increase/decrease in recall/sensitivity observed for the specific groups of liquids. Still, the fact that the performances of the RF (and to a lesser extent the *k*NN) algorithm improved to DL levels suggests that in the present case, models’ performances are more affected by the size of the dataset used for model training rather than by the nature of the ML algorithm. Finally, while we cannot rule out the possibility that (the small) improvements in performances of models trained on the real+synthetic data resulted from similarity between training and test sets (the synthetic spectra were generated based on the real spectra from the training set), we note that all models performed well on the second dataset whose spectra were not used for spectra synthesis.

The main conclusions of this study are as follows. (1) It is possible to derive classification models that will reliably differentiate between petroleum distillates, gasoline, and a complementary group containing other types of flammable liquids based on their GS spectra. In particular, we note the good performances of the *k*NN and RF models which were trained and validated on real data and therefore do not suffer from any biases that may have been introduced by our spectra synthesis algorithm. (2) For this specific application, ‘simpler’ models (in particular *k*NN and RF) often perform as well as more complex models (DL), provided they were trained on datasets of similar size. (3) In agreement with previous observations [10–17], model derivation does not necessarily require a database of millions of real samples. Rather, a set of synthetic data, carefully derived from physical principle could be used. This is also true for algorithms that inherently require a large dataset for their training (e.g., DL). This observation may open the door for the usage of powerful, DL‐based methods for small datasets, provided that appropriate physical principles could be identified for the derivation of reliable synthetic data.

We envision that the present work could evolve in several directions. First, it could be readily extended to the classification of other flammable liquids. Importantly, the spectra synthesis algorithm developed in this work paves the way towards applications to liquids that are less commonly used in arsons cases. Second, other data synthesis algorithms could be tested. Third, the workflow could be used to identify the sample size of real data required to produce reliable models. Finally, model interpreters (e.g., SHAP values [28]) could be used to understand, for example, why a sample was classified the way it was. Finally, on a more global level, this work could be extended to other forensic domains which characterize samples by spectra either solely or in combination with other techniques and by extension, to additional fields.

## Author Contributions

**Omer Kaspi:** study design, data curation, code writing, data analysis and interpretation, writing and editing. **Yaniv Y. Avissar:** data collection and curation, data analysis and interpretation, editing. **Arnon Grafit:** data collection and curation, data analysis and interpretation. **Ron Chibel:** project supervision, data analysis and interpretation. **Olga Girshevitz:** data analysis and interpretation. **Hanoch Senderowitz:** project supervision, study design, data analysis and interpretation, writing and editing, funding.

## Conflicts of Interest

The authors declare no conflicts of interest.

## Data Availability

The data that support the findings of this study are available from the corresponding author upon reasonable request.
